# Acute Diphtheritic Whitlow

**Published:** 1904-12-24

**Authors:** 


### ACUTE DIPHTHERITIC WHITLOW.

As secondary affections, whitlows, due to the bacilli of diphtheria, are not uncommon in cases of diphtheria, and they rarely lead to any serious trouble. But it is well to remember that they may occur as primary conditions in people who apparently are in other ways in good health, and in these cases it is very necessary that the true cause should be recognised, both for the safety of the patient and those around him. In the current number of the

St. Bartholomew's Hospital *Journal* Dr. G. C. Garratt describes three cases. The first was a girl of five years, convalescent from scarlet fever. A large bulla containing opalescent fluid developed on the side of one index finger. The epithelium was snipped off with scissors and lead lotion was applied to the raw surface. But the sore showed no sign of healing and had a tendency to bleed. Cultivations showed the presence of Klebs-Lœffler bacilli, and after an injection of anti-toxin had been given the sore rapidly healed. The temperature was subnormal throughout. The second case was that of a child of two years, and, like the first, she was convalescent from scarlet fever. One of her thumbs became red and tender and next day a bleb, from which thin pus was evacuated, formed on it. The sore was treated with fomentations, but the inflammation spread, the back of the hand became œdematous, and there was lymphangitis of the forearm. An incision was made down to the bone, and, in addition, the nail was removed, but no pus was found. The wound showed no tendency to heal. On the seventh day from the commencement of the inflammation the child vomited several times. A cultivation was now made, and diphtheria bacilli were found. A dose of antitoxin was therefore administered on the ninth day. The following night the child suddenly sat up, vomited, and fell back dead. In the third case the patient was a woman aged 54. She came for treatment on account of a large bulla with surrounding inflammation on her thumb. Blood-stained serum escaped when the bulla was opened. Next day, in spite of a dry antiseptic dressing, a ring of vesicles had formed around the sore left by the primary bulla, and the hand was œdematous. Diphtheria was now suspected, and inquiry elicited the fact that three weeks earlier the patient had suffered from a severe sore throat accompanied by blood-stained discharge from the nose. Swabs were made both from the nose and hand, and Klebs-Lœffler bacilli were grown from them. After the administration of antitoxin the case progressed favourably, though there was some sloughing which necessitated amputation of the affected thumb. The temperature was subnormal throughout except for two temporary rises.

In commenting on these cases Dr. Garratt points out that in diphtheritic whitlow as in faucial diphtheria the actual destruction of tissue is small and comparatively superficial, in spite of the severity of the local lesion. The points which should lead to a suspicion of diphtheria in a case of whitlow are the presence in the superficial lesion of very thin pus or serum only, especially if bloodstained, the absence of deep suppuration, the tendency to spread by the skin and subcutaneous tissues rather than along the tendon sheaths, the presence of a soft doughy œdema, and in particular a temperature remaining subnormal in spite of the severe local inflammation. The prevalence of diphtheria in the district, the history of sore throat or the presence of rhinorrhœa would afford strong confirmatory evidence; but a culture will definitely settle the question.

